# Ultra-thin porous calcium phosphate coatings show extensive remodeling and high osseointegration after 24 weeks in a rabbit femoral condyle model

**DOI:** 10.3389/fbioe.2026.1966955

**Published:** 2026-09-22

**Authors:** Mihail Genchev, Bianca Riedel, Coralie Nagels, Hagen Schmal, Eva Johanna Kubosch, Maria Carolina Lanzino, Andreas Killinger, Sofia Dembski, Anika Höppel, Michael Seidenstuecker

**Affiliations:** 1 G.E.R.N. Research Center for Tissue Replacement, Regeneration & Neogenesis, Department of Orthopedics and Trauma Surgery, Medical Center-Albert-Ludwigs-University of Freiburg, Faculty of Medicine, Albert-Ludwigs-University of Freiburg, Freiburg, Germany; 2 Department of Orthopedics and Trauma Surgery, Medical Center-Albert-Ludwigs-University of Freiburg, Faculty of Medicine, Albert-Ludwigs-University of Freiburg, Freiburg, Germany; 3 Department of Orthopedic Surgery and Traumatology, Odense University Hospital, Odense, Denmark; 4 Institute for Ceramic Materials and Technologies (IKMT), University of Stuttgart, Stuttgart, Germany; 5 Fraunhofer Institute for Silicate Research ISC, Würzburg, Germany; 6 Department for Functional Materials in Medicine and Dentistry, University of Würzburg, Würzburg, Germany; 7 Technical University of Applied Sciences Würzburg-Schweinfurt, Faculty of Electrical Engineering, Schweinfurt, Germany

**Keywords:** calcium phosphate, copper functionalization, GB14, histomorphometry, osseointegration, ultra-thin porous coatings, β-tcp

## Abstract

Ultra-thin porous calcium phosphate (CaP) coatings may provide a transient osteoconductive interface while reducing the amount of coating material remaining on an implant. However, it remains unclear whether extensive remodeling of such coatings is compatible with high osseointegration *in vivo.* In this study, approximately 30-µm-thick porous high-velocity suspension flame-sprayed β-tricalcium phosphate (β-TCP) and GB14 (Ca_2_KNa(PO_4_)_2_) coatings, with and without copper-functionalized supraparticles, were evaluated in a rabbit femoral condyle model. The coatings were deposited onto TiAl6Nb7 implants and characterized with respect to morphology, thickness, porosity, and surface roughness. After 24 weeks *in vivo,* 19 evaluable implants were assessed by qualitative histology, quantitative histomorphometry, and sequential fluorochrome labeling. No histological signs of inflammatory tissue reactions were observed, and all coating variants showed direct bone apposition without interposed connective tissue. Bone-to-implant contact ranged from 76.5% ± 14.2% to 86.3% ± 4.6%, whereas residual coating coverage accounted for only 1.5% ± 1.8% to 6.4% ± 6.8% of the implant perimeter. Fluorochrome incorporation at 2, 4, 6, and 8 weeks indicated peri-implant bone formation at all investigated labeling time points. Paired analyses revealed no statistically significant Cu-associated differences in the investigated cellular, histomorphometric, or fluorochrome-labeling outcomes within either CaP system, either before or after false-discovery-rate correction. These findings demonstrate the coexistence of extensive coating remodeling and high osseointegration at 24 weeks across two ultra-thin porous CaP coating systems. The low residual coating coverage together with direct bone apposition supports the concept of ultra-thin bioresorbable CaP coatings as transient osteoconductive interfaces for orthopedic implants.

## 1 Introduction

The increasing number of primary and revision joint arthroplasties has intensified the need for implant surfaces that support rapid and durable biological fixation (Matharu et al., 2022; Hauer et al., 2024). Aseptic and septic loosening nevertheless remain major causes of implant failure and revision surgery, with many of the underlying processes originating at the bone-implant interface (Hauer et al., 2024; Hodges et al., 2021). Implant failure is multifactorial, and processes at the coated interface act alongside substrate-related mechanisms such as stress shielding caused by the elastic-modulus mismatch between metallic implants and surrounding bone (Niinomi, 1998). Calcium phosphate (CaP) coatings are widely used to improve the biological fixation of metallic implants because of their chemical similarity to the mineral phase of bone and their osteoconductive properties (Nuss and von Rechenberg, 2008; Grzeskowiak et al., 2020; Heimann, 2017). Plasma-sprayed hydroxyapatite has consequently become an established clinical coating for cementless orthopedic implants (Heimann, 2017; Botterill et al., 2022). However, conventional CaP coatings may be affected by residual stresses, interfacial delamination, particulate release, and heterogeneous or incomplete degradation, potentially compromising long-term implant performance (Heimann, 2017; Wang et al., 1993; Morscher et al., 1998; Schönweger et al., 2020). Increasing coating porosity can promote biological remodeling but may simultaneously reduce mechanical integrity, illustrating the balance required between osteoconductivity, resorption, and coating stability (Heimann, 2017; Wang et al., 1993; Morscher et al., 1998; Schönweger et al., 2020). Reducing coating thickness while retaining a porous and osteoconductive surface represents a complementary strategy for balancing biological activity and coating stability. High-velocity suspension flame spraying (HVSFS) enables the deposition of homogeneous porous CaP coatings with a thickness of approximately 30 μm, substantially below the 150–250 µm commonly reported for conventional plasma-sprayed CaP coatings (Heimann, 2017; Genchev et al., 2026a). Whether this reduced coating thickness provides biological or mechanical advantages over conventional-thickness coatings requires direct comparative evaluation. Rather than functioning as a permanent interfacial layer, such ultra-thin bioresorbable coatings are intended to provide a transient osteoconductive surface during early bone healing and subsequently be replaced by newly formed bone (Burtscher et al., 2019; Subkov et al., 2025). Previous physicochemical, *in vitro,* and biomechanical investigations demonstrated that these HVSFS coatings possess favorable coating characteristics, cytocompatibility, and implant fixation (Lanzino et al., 2024a; Genchev et al., 2026a; Le et al., 2025). However, their low material volume also raises an important question: whether extensive loss of the coating during *in vivo* remodeling remains compatible with direct bone apposition and high bone-implant contact. To investigate whether this remodeling concept is applicable across different CaP compositions, two bioresorbable coating materials were selected. β-Tricalcium phosphate (β-TCP) served as an established reference material because of its osteoconductivity and predictable *in vivo* degradation (Bernstein et al., 2008; Lu et al., 2018; Wiltfang et al., 2002). GB14 (Ca_2_KNa(PO_4_)_2_), a potassium- and sodium-containing calcium phosphate, was included as an alternative composition with distinct physicochemical properties and ion-release characteristics (Lanzino et al., 2024a; Berger et al., 1995). Both materials can be processed into porous ultra-thin coatings by HVSFS and have previously shown favorable physicochemical, mechanical, and biological properties (Lanzino et al., 2024a; Genchev et al., 2026a; Le et al., 2025). Each coating system was additionally investigated with and without copper-functionalized supraparticles. Copper was selected because of its reported osteogenic, angiogenic, and antimicrobial properties (Barralet et al., 2009; Pierre et al., 2023; Han et al., 2019; Jacobs et al., 2021), while previous *in vitro* studies demonstrated that the employed supraparticle-based functionalization can provide antibacterial activity without compromising cytocompatibility (Le et al., 2025; Höppel et al., 2024; Lanzino et al., 2024b). The supraparticle fraction used in the present study was retained from this previously characterized formulation rather than selected through an additional *in vivo* dose-finding experiment. This design enabled paired assessment of copper functionalization within each CaP system, whereas the two CaP compositions were investigated in separate animal cohorts and were therefore not intended for direct inferential comparison. Accordingly, the primary aim of this study was to determine whether extensive remodeling of approximately 30-µm-thick porous CaP coatings remains compatible with high osseointegration after 24 weeks *in vivo.* HVSFS β-TCP and GB14 coatings, each with and without copper-functionalized supraparticles, were evaluated in the distal femoral condyles of New Zealand White rabbits using qualitative histology, quantitative histomorphometry, and sequential fluorochrome labeling. Residual coating coverage and bone-implant contact were assessed as the principal measures of coating remodeling and osseointegration, respectively. Secondary analyses examined whether CaP composition or copper functionalization influenced peri-implant cell density, bone area, and fluorochrome-labeled bone formation. The specific contribution of the present study is the combined quantitative assessment of residual coating coverage, bone-to-implant contact, peri-implant bone area, cell density, and sequential fluorochrome labeling after 24 weeks. Previous publications addressed the physicochemical and *in vitro* properties of related HVSFS coatings, the histological performance of earlier coating formulations, or the biomechanical fixation of complementary specimen portions from the same animal experiment. The present analysis adds endpoint-specific histological evidence showing that minimal residual coating coverage can coexist with high BIC across both investigated ultra-thin CaP coating systems. We hypothesized that all coating variants would exhibit low residual coating coverage together with direct bone apposition and high bone-to-implant contact, whereas copper functionalization might modulate selected parameters of the peri-implant bone response within each CaP system.

## 2 Materials and methods

β-TCP (Budenheim, Germany), GB14 (Federal Institute for Materials Research and Testing, Germany), and TiAl6Nb7 rods (DePuy Synthes, USA) were used as coating materials and substrates. Chemicals for histological processing were obtained from commercial suppliers and used as received unless otherwise stated.

### 2.1 Coating fabrication and specimen preparation

Ultra-thin porous CaP coatings were deposited onto TiAl_6_Nb_7_ rods (240 mm × 4 mm) via high-velocity suspension flame spraying (HVSFS). Suspensions based on β-tricalcium phosphate (β-TCP) and GB14 (Ca_2_KNa(PO_4_)_2_) were prepared at 5 wt% solid loading in deionized water and stabilized using a hydrocolloid (2 wt%) and a phosphonate-based dispersant (3 wt% relative to solids). For Cu-functionalized coatings, 0.5 wt% Cu-doped supraparticles (5 wt% Cu) were added in a secondary mixing step. This loading was selected based on previous physicochemical and *in vitro* investigations of the same supraparticle-functionalized coating formulation, which demonstrated Cu release, antibacterial activity, and acceptable cytocompatibility (Le et al., 2025; Höppel et al., 2024; Lanzino et al., 2024b). Because an already characterized formulation was investigated, no additional *in vivo* dose-ranging groups were included, thereby limiting animal use in accordance with the 3R principles. Prior to coating, substrates were grit blasted (F60 corundum, 4 bar; Sa ≈2.7 µm) and ultrasonically cleaned in acetone. Coatings were applied using a robot-guided HVSFS system with a defined meander trajectory. Spraying distances (120 mm for β-TCP, 140 mm for GB14) were adopted from prior optimization to ensure reproducible coating characteristics. Coated rods were segmented into cylindrical implants approximately 16 mm in length. A total of 24 implants were used for the *in vivo* study, comprising six implants for each coating formulation (β-TCP, β-TCP/Cu, GB14, and GB14/Cu). Additional coated rod segments were retained for physicochemical characterization. Representative segments approximately 15 mm in length were used for X-ray diffraction and supplementary coating analyses. Prior to implantation, the coated implants were sterilized by gamma irradiation at a dose of 10 kGy (BBF Sterilisationsservice GmbH, Kernen, Germany).

### 2.2 Coating characterization

Coating morphology and microstructure were evaluated by field-emission scanning electron microscopy (SEM; S-800, Hitachi High-Technologies Corporation) using backscattered electron imaging on carbon-coated cross-sections, and additionally by optical microscopy (MeF4M, Leica GmbH) in bright field. Acquired images were analyzed using ImageJ and Olympus analySIS software. Porosity was quantified from SEM micrographs (×1400, 10 kV) by converting images to 8-bit format and applying uniform threshold segmentation to distinguish pore regions, followed by binary particle analysis; values represent the mean of three images per sample. Coating thickness was determined in accordance with DIN EN ISO 1463:2021–08 by measuring fifteen individual thickness values per sample and calculating mean values and standard deviations. Phase composition of representative HVSFS coatings had been characterized previously by X-ray diffraction (Lanzino et al., 2024a; Subkov et al., 2025) using Cu-Kα radiation (λ = 0.1541 nm) over a 2θ range of 20°–70° (step size, 0.02°; acquisition time, 5 s/step) on coated rod segments approximately 15 mm in length. These previous analyses were not designed as paired pre- and post-sterilization measurements, and the oxidation state of copper was not assessed. Areal surface roughness parameters (Sa and Sz) were determined using a 3D laser scanning microscope (VK-X250, Keyence, Osaka, Japan). For each coating formulation, five independent samples were analyzed at ×400 magnification, with ten standardized surface areas of 100 × 100 µm measured per sample. The ten measurements were averaged for each sample, and results are reported as mean ± standard deviation across the five samples. The release behavior of the same 0.5 wt% supraparticle-functionalized coating formulation had previously been evaluated in TRIS buffer over 120 h, showing an initial release within the first 24 h followed by a progressively decreasing release rate (Lanzino et al., 2024a; Subkov et al., 2025). Local Cu concentrations and *in vivo* release kinetics were not measured in the present animal experiment.

### 2.3 Animal model and surgical procedure

The animal model, including implantation, harvesting, and specimen preparation, has been described previously (Genchev et al., 2026a). Briefly, twelve skeletally mature female New Zealand White rabbits (≥26 weeks of age; body weight, 3.75 ± 0.16 kg) were included in the study. Female animals were selected to maintain comparability with previous studies using the same rabbit model. All procedures were approved by the Regional Council of Freiburg (G-23/079) and conducted in accordance with §8 TierSchG, Directive 2010/63/EU, and ISO EN 10993–2:2023–02. Animals were allocated to two material cohorts comprising six rabbits each: a β-TCP cohort and a GB14 cohort. Each animal received two implants of the same CaP composition, with the copper-functionalized coating implanted in the left femoral condyle and the corresponding copper-free coating implanted in the right femoral condyle. This resulted in four coating groups with six implants each (β-TCP, β-TCP/Cu, GB14, and GB14/Cu). Copper effects were therefore assessed using paired implants within the same animal, whereas comparisons between β-TCP and GB14 involved independent animal cohorts. All GB14-coated implants were placed during the first surgical session and all β-TCP-coated implants during the second session using the same standardized surgical protocol. Implant side was fixed rather than randomized to standardize the surgical workflow and prevent confusion between copper-functionalized and copper-free implants. Before submission of the animal-study application, an *a priori* sample-size assessment was performed by the Institute of Medical Biometry and Statistics, University Medical Center Freiburg. The overarching exploratory animal experiment defined biomechanical push-out force and the histological extent of new bone formation as primary outcomes. The sample-size calculation was driven by the biomechanical endpoint and assumed a standardized effect size of 1.8 based on previous experimental experience. For a two-sided independent-samples t-test with α = 0.05 and 80% statistical power, six animals per experimental condition were required. Histological bone formation was intended primarily as an estimative outcome and was expected to exhibit comparatively low variability. The paired comparison between Cu-functionalized and Cu-free coatings was defined as secondary and was not used to determine the sample size. Accordingly, six animals were included in each material cohort. No uncoated titanium, conventional-thickness CaP, or Cu-only control groups were included. Uncoated implants and conventional plasma-sprayed hydroxyapatite coatings had previously been evaluated alongside rapidly resorbable CaP coatings in a related rabbit model by Bernstein et al. (Bernstein et al., 2008). The present experiment therefore focused on paired comparisons between Cu-functionalized and Cu-free variants of the same ultra-thin CaP coating, thereby limiting the number of additional animals in accordance with the 3R principles. A separate Cu-only group was not included because the investigated functionalization consisted of Cu-doped CaP supraparticles embedded within the respective CaP coating matrix rather than a stand-alone copper surface. Consequently, the present design does not permit direct determination of whether the observed interface differs from uncoated titanium or conventional-thickness coatings, or whether any Cu-associated response depends on the surrounding CaP matrix. Surgical implantation was performed under sterile conditions using a standardized protocol. General anesthesia was induced and maintained using a combination of ketamine, medetomidine, and isoflurane, supplemented by systemic and local analgesia. Following lateral access to the distal femoral condyle, a cylindrical defect (4.0 mm) was created by drilling, and coated implants were press-fitted using an applicator system (ActivaPin®, Bioretec). Wounds were closed in layers, and postoperative analgesia was administered. Animals were allowed unrestricted weight bearing pre- and postoperatively. To monitor the temporal progression of bone formation, four different fluorochrome labels were administered by subcutaneous injection. Tetracycline and xylenol orange (both Sigma-Aldrich, now Merck, Darmstadt, Germany), together with calcein and alizarin complexone (both Carl Roth GmbH + Co. KG, Karlsruhe, Germany), were prepared in a 2% aqueous NaHCO_3_ solution (Sigma-Aldrich, now Merck, Darmstadt, Germany), allowing an injection volume of 1 mL per animal (Genchev et al., 2026a). Each fluorochrome was administered as a single injection at 2, 4, 6, and 8 weeks postoperatively. After a healing period of 24 weeks, all animals were euthanized under deep anesthesia. Analgosedation was induced by subcutaneous administration of ketamine (20 mg/kg body weight) and midazolam (0.3 mg/kg body weight). Subsequently, a 22-G intravenous catheter was placed in the marginal ear vein, and propofol (5–10 mg/kg body weight, i.v.) was administered to induce deep anesthesia. Euthanasia was then performed by intravenous administration of potassium chloride (2 mmol/kg body weight).

#### 2.3.1 Implant harvesting and specimen preparation

Following euthanasia, femora were dissected, and the distal segments containing the implants were isolated and fixed in 70% ethanol. Each implant-containing metaphysis was sectioned longitudinally using a diamond band saw (Walther Messmer GmbH, Oststeinbek, Germany) under controlled conditions to preserve the bone-implant interface. A custom holder ensured perpendicular sectioning relative to the implant axis, minimizing mechanical artifacts (Genchev et al., 2026b). The lateral specimen halves were sectioned into standardized 2-mm-thick slices for push-out testing, the results of which have been reported previously (Genchev et al., 2026a). The corresponding specimen halves were retained for the histological and histomorphometric analyses presented in the current study.

### 2.4 Histological processing and histomorphometric analysis

Bone preparation was performed as previously described by Genchev et al. (Genchev et al., 2026a). A dedicated holding device was used to ensure sectioning of the implant in the desired plane and to improve the quality and reproducibility of the histological specimens (Genchev et al., 2026a; Genchev et al., 2026b). The embedding procedure followed protocols previously reported by Bernstein et al. and Subkov et al. (Subkov et al., 2025; Bernstein et al., 2008). Following initial fixation and storage in 70% ethanol, the femoral condyle specimens were trimmed to include the predefined region of interest. At the beginning of the embedding protocol, the specimens were rinsed and transferred to 40% ethanol for 1 day. They were subsequently dehydrated using a graded ethanol series comprising 70%, 80%, 96%, and 100% ethanol, cleared in xylene, and embedded in polymethyl methacrylate (PMMA), as detailed in Table 1. To preserve the integrity of the bone-implant interface, undecalcified sections were subsequently prepared using the cutting-grinding technique described by Donath (1988) (Donath, 1988). Embedding was performed initially in pure MMA, followed by infiltration with an MMA mixture containing benzoyl peroxide (BPO, Merck) and dibutyl phthalate (DBP, Alfa Aesar, Heysham, UK). After polymerization, the embedded tissue blocks were sectioned perpendicular to the longitudinal implant axis using a Secotom-50 precision cutting machine equipped with a 200-µm-thick cutting disc (Struers, Ballerup, Denmark). Histological sections were considered technically non-evaluable when insufficient staining or air-bubble artifacts introduced during methyl methacrylate embedding prevented reliable identification and quantification of the peri-implant tissue or bone-implant interface. Sections approximately 300 µm thick were obtained, mounted on PMMA slides (AbaMediCus, Halle, Germany), and subsequently ground to a final thickness of approximately 100 µm using progressively finer abrasive papers up to 4,000 grit. The white PMMA slides provided a consistent background and optimized scattered-light contrast during microscopy. Histological evaluation and image acquisition were performed using an Olympus BX53 microscope (Olympus Co. Ltd., Tokyo, Japan).

**TABLE 1:** Protocol for PMMA embedding.

Reagent	Days
40% EtOH	1
70% EtOH	3
80% EtOH	3
96% EtOH	2
100% EtOH I	2
100% EtOH II	2
Xylol I	1
Xylol II	2
MMA	5
MMA MMA+1%BPO+5%DBP	3
Polymerisation MMA+3%BPO+5%DBP	21

#### 2.4.1 Histology

Histological evaluation was performed as previously described by Subkov et al. (Subkov et al., 2025). Giemsa staining was performed for subsequent histological and histomorphometric analyses. High-resolution images of the regions of interest (ROIs) were acquired using an Olympus BX53 microscope and archived as JPEG files. To ensure reproducibility across all ground sections, a digital polygon template was generated using Adobe Photoshop CS6 64-bit. The template was aligned with the longitudinal axis of the bone, resulting in six standardized ROIs per specimen. Within each primary ROI, a secondary digital frame calibrated to the field of view at ×20 magnification was superimposed to define the specific analytical area. This frame was positioned in a standardized manner to ensure equal representation of the coated titanium implant and the adjacent peri-implant bone at a 1:1 ratio. The standardized ROIs were assessed by bright-field microscopy at ×20 magnification. Exposure time was kept constant for all images, whereas the focus was adjusted individually to compensate for minor variations in final section thickness.

#### 2.4.2 Cell counting

For cell counting, images were acquired at ×20 magnification using an Olympus BX53 microscope (Olympus Co. Ltd., Tokyo, Japan). Cell quantification was performed using the open-source image analysis software Fiji (version 1.54p 17). For each specimen, six standardized regions of interest (ROIs) were analyzed, resulting in a total of 114 ROIs across 19 specimens. Osteocytes were identified as cells embedded within lacunae in the mineralized bone matrix. Osteoblasts were identified as mononuclear cells lining bone surfaces, whereas osteoclasts were identified as large multinucleated cells located at bone surfaces, frequently within resorption lacunae. Automated detection was based on predefined thresholds for staining intensity, object size, and morphology, together with the characteristic anatomical location of each cell type. All automated classifications were subsequently verified by manual inspection and corrected where necessary. The effective tissue area within each ROI was measured using Fiji. Cell density was calculated by normalizing the number of counted cells to the corresponding tissue area and was expressed as cells per mm^2^. The cell densities obtained from the six ROIs were averaged for each specimen, and this specimen-level mean was used as the independent value in all subsequent statistical analyses. The workflow for ROI selection and cell quantification is illustrated in Figure 1. The evaluator performing the manual verification and correction of the automated cell classifications was not formally blinded to the coating groups. Nevertheless, all specimens were assessed using the same predefined cell-identification criteria, standardized ROI templates, and analysis settings. No formal intra- or inter-observer agreement assessment was performed.

**FIGURE 1:**
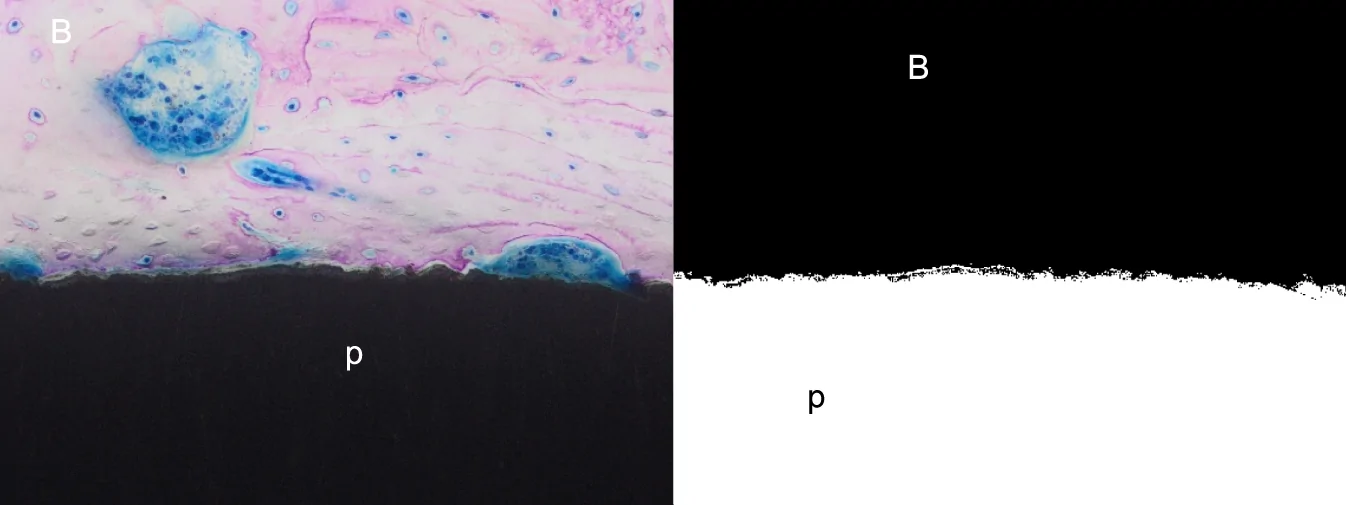
Representative microscopic image used for cell counting and the corresponding mask applied for calculation of the effective tissue area. B- Bone; p - pin.

#### 2.4.3 Histomorphometry

For histomorphometric analysis, one Giemsa-stained section was randomly selected from each specimen. Histological sections were examined using an Olympus BX53 microscope. To capture the entire implant perimeter, including the surrounding peri-implant tissue, up to 72 individual images were acquired at ×10 magnification and subsequently merged into a composite image using Olympus Stream Motion software.

##### 2.4.3.1 Bone to implant contact

Bone-implant contact (BIC) was quantified using Adobe Photoshop CS6 and Fiji (Figure 2). The beginning and end of each implant-perimeter segment without direct contact between bone and the implant or residual coating were manually marked. Using the center of the circular implant as the vertex, the angular extent of each non-contact segment was measured. The angles of all non-contact segments were summed, and BIC was calculated relative to the complete implant circumference according to:

**FIGURE 2:**
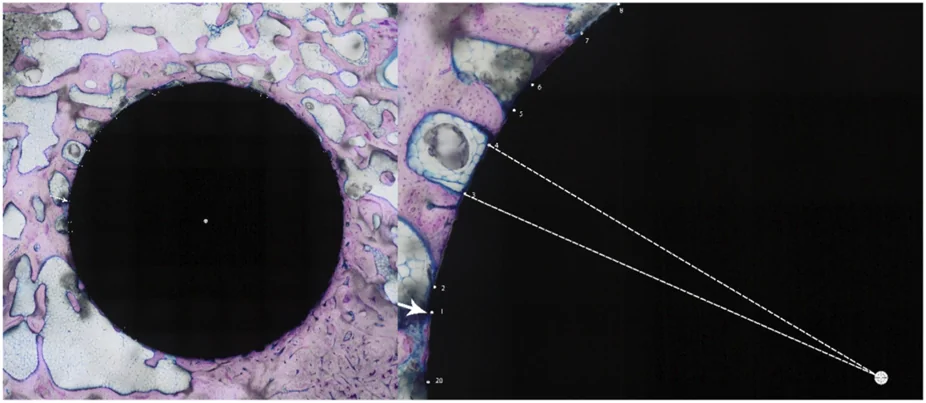
Illustration of the angle-based method used to quantify the bone-implant contact surface. In this representative example, the angle between two adjacent reference points bordering an area without direct bone-implant contact is shown. This angular segment was used to calculate the non-contact fraction relative to the total implant circumference.

BIC (%) = [1 − (Σθ_non-contact/360°)] × 100.

Direct contact between bone and either the exposed implant surface or residual coating was considered bone-implant contact.

##### 2.4.3.2 Residual coating coverage

Residual coating coverage was quantified from the same composite histological images using an angle-based approach. The beginning and end of each implant-perimeter segment containing histologically identifiable residual coating material were manually marked. Using the implant center as the vertex, the angular extent of each coated segment was measured. The angles of all segments containing residual coating were summed, and residual coating coverage was calculated relative to the complete implant circumference according to:

Residual coating coverage (%) = (Σθ_residual coating/360°) × 100.

The evaluators performing the manual identification of contact and non-contact regions and of residual coating segments were not formally blinded to group allocation. Standardized composite images, predefined geometric criteria, and the same angle-based measurement procedure were applied to all specimens. No formal intra- or inter-observer agreement data were obtained.

##### 2.4.3.3 Bone area

Peri-implant bone area was quantified within a circumferential region of interest extending 0.5 mm outward from the implant surface. The ROI was delineated in Adobe Photoshop CS6 and subsequently analyzed using Fiji (version 1.54p17). Images were converted into binary masks to distinguish bone from non-bone tissue. Because staining intensity varied between sections, the segmentation threshold was adjusted individually for each image to exclude the unstained white background while retaining the stained bone tissue. The resulting binary masks were visually verified against the corresponding histological images before quantification. Peri-implant bone area was calculated as the segmented bone area relative to the total area of the 0.5-mm circumferential ROI and expressed as a percentage, as illustrated in Figure 3.

**FIGURE 3:**
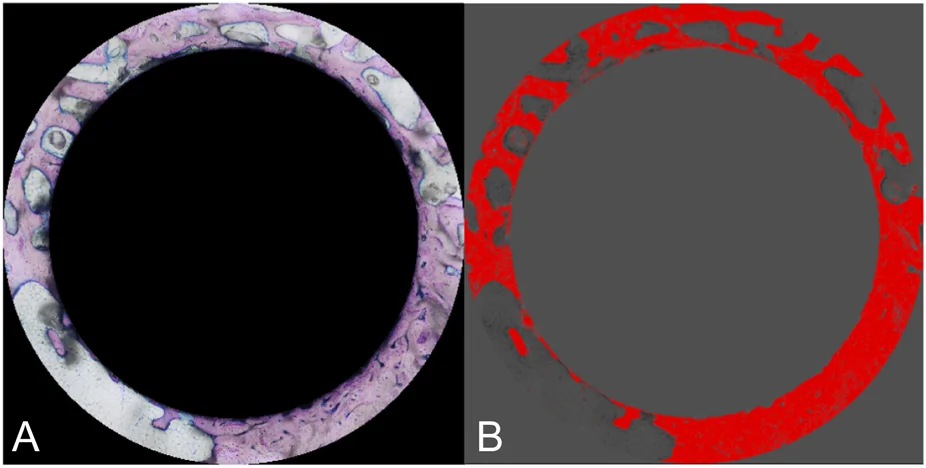
Quantification of peri-implant bone area. **(A)** The 0.5-mm peri-implant ring was defined as the region of interest (ROI) for measurement of the surrounding bone area. **(B)** Corresponding binary mask of the ROI. The red area represents bone tissue used for quantitative analysis.

### 2.5 Fluorochrome sequential labelling

To visualize peri-implant bone repair, fluorochrome sequential labelling was performed. For dynamic histomorphometric assessment of bone formation, the animals received sequential subcutaneous injections of four fluorochrome markers according to the protocols described by Rahn (Rahn, 1976) and Bernstein (Bernstein, 2007). Tetracycline was administered 2 weeks postoperatively at a dose of 25 mg/kg. Xylenol orange was injected at 4 weeks postoperatively at a dose of 80 mg/kg, followed by calcein at 6 weeks postoperatively at 10 mg/kg and alizarin complexone at 8 weeks postoperatively at 30 mg/kg. Specimen preparation was performed as described above. Fluorescence images of the different fluorochrome labels were acquired at ×20 magnification using a Olympus BX51 fluorescence microscope (Olympus Corporation, Tokyo, Japan). For each fluorochrome channel, exposure time and all other acquisition settings were kept constant across specimens. A fixed channel-specific threshold was applied to all images when generating the binary masks. For each specimen, four standardized peri-implant ROIs were analyzed. The individual fluorescence channels were converted into binary masks using Fiji (version 1.54p17), and the fluorochrome-positive area was measured separately for each label. The labeled area fraction was calculated as the fluorochrome-positive area relative to the total ROI area. For each fluorochrome, the values obtained from the four ROIs were averaged for each specimen, and this specimen-level mean was used as the independent value in subsequent statistical analyses. The segmentation workflow is illustrated in Figure 4.

**FIGURE 4:**
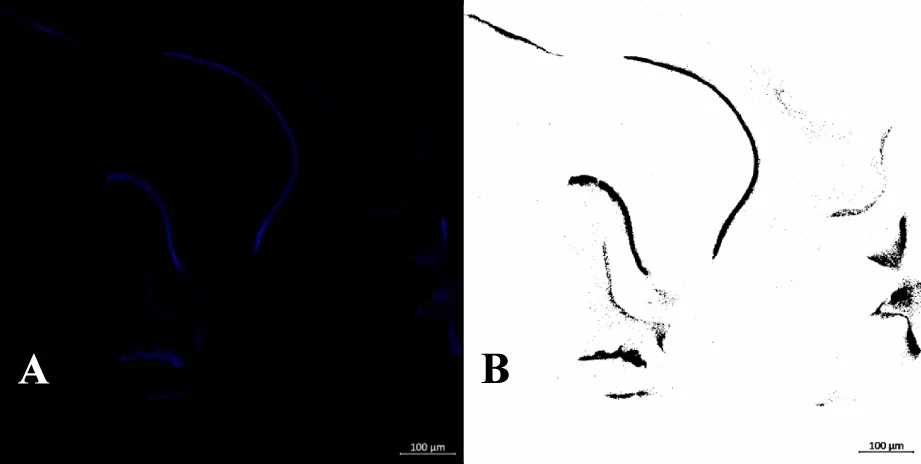
Quantification of fluorochrome-labeled bone formation. **(A)** Representative fluorescence image of the peri-implant bone region. **(B)** Corresponding binary mask illustrating the segmented fluorescent area used for quantitative analysis.

### 2.6 Statistical analysis

Data are presented as mean ± standard deviation; median and interquartile range values were additionally considered for outcomes analyzed using non-parametric tests. For cell density, peri-implant bone area, bone-to-implant contact, residual coating coverage, and fluorochrome-positive area fraction, ROI-level measurements were first averaged for each specimen, and the resulting specimen-level means were used as the independent values for statistical analysis. Copper-functionalized and copper-free coatings were compared separately within each CaP system using paired-samples tests, reflecting the bilateral implantation design. Normality of the within-animal differences was assessed using the Shapiro–Wilk test. Normally distributed paired differences were analyzed using a two-sided paired-samples t-test; otherwise, a two-sided Wilcoxon signed-rank test with the exact p-value was applied. For the GB14 cohort, only the four animals with evaluable bilateral specimens were included in paired comparisons. The additional evaluable GB14 implant was retained for descriptive statistics only. Pooled β-TCP/all and GB14/all values were presented descriptively in selected tables and figures but were not treated as independent groups or subjected to inferential comparison. Nine Cu-versus-control comparisons were evaluated within each CaP system: osteocyte density, combined osteoblast/osteoclast density, peri-implant bone area, bone-to-implant contact, residual coating coverage, and the four fluorochrome-positive area fractions. To account for multiple testing, the resulting p-values were adjusted separately within each CaP system using the Benjamini–Hochberg false-discovery-rate procedure. Raw two-sided p-values and FDR-adjusted p-values are reported, with statistical significance defined as p_FDR_ < 0.05. Statistical analyses were performed using Origin 2026 Professional SR1 (OriginLab Corporation, Northampton, MA, USA).

## 3 Results

Of the 24 implanted specimens, 19 were available for final analysis. One animal from the β-TCP cohort developed a postoperative infection and was euthanized prematurely after reaching the predefined humane endpoint of more than 20% body-weight loss within 3 days. Consequently, both implants from this animal were excluded. Three additional specimens from the GB14 cohort—one GB14 and two GB14/Cu implants—were technically non-evaluable because insufficient staining and/or air-bubble artifacts introduced during methyl methacrylate embedding prevented reliable histological and histomorphometric evaluation. The final group sizes were therefore n = 5 for β-TCP, n = 5 for β-TCP/Cu, n = 5 for GB14, and n = 4 for GB14/Cu, providing five complete bilateral pairs in the β-TCP cohort and four complete bilateral pairs in the GB14 cohort. The additional evaluable GB14 implant without a corresponding evaluable GB14/Cu specimen was retained for descriptive presentation only. For descriptive presentation, β-TCP and β-TCP/Cu were additionally summarized as β-TCP/all, whereas GB14 and GB14/Cu were summarized as GB14/all. These pooled categories were not included as independent groups in inferential statistical analyses. An overview of the group designations is provided in Table 2.

**TABLE 2:** Experimental coating groups and abbreviations used in tables and figures.

Group	Composition	Label in figures
β-TCP/all	Pooled β-TCP and β-TCP/Cu specimens	TCP/all
β-TCP	β-TCP without copper functionalization	TCP
β-TCP/Cu	Copper-functionalized β-TCP	TCP/Cu
GB14/all	Pooled GB14 and GB14/Cu specimens	GB14/all
GB14	GB14 without copper functionalization	GB14
GB14/Cu	Copper-functionalized GB14	GB14/Cu

### 3.1 Coating characterization

The physicochemical characterization of the ultra-thin porous HVSFS CaP coatings has been reported previously as part of the biomechanical study using complementary portions of specimens from the same animal experiment (Genchev et al., 2026a). Briefly, all four ultra-thin coating formulations exhibited a porous microstructure, with mean coating thicknesses ranging from 27.9 ± 2.5 to 31.5 ± 2.4 µm and porosities ranging from 13.1% ± 1.6% to 14.3% ± 1.0%. Mean Sa values ranged from 2.4 ± 0.1 to 3.1 ± 0.4 µm. No marked differences in these coating characteristics were observed between the formulations. Representative cross-sectional SEM images and selected physicochemical characteristics are reproduced in Figure 5 and Table 3 under the applicable CC BY 4.0 license solely to contextualize the histological and histomorphometric findings of the present study.

**FIGURE 5:**
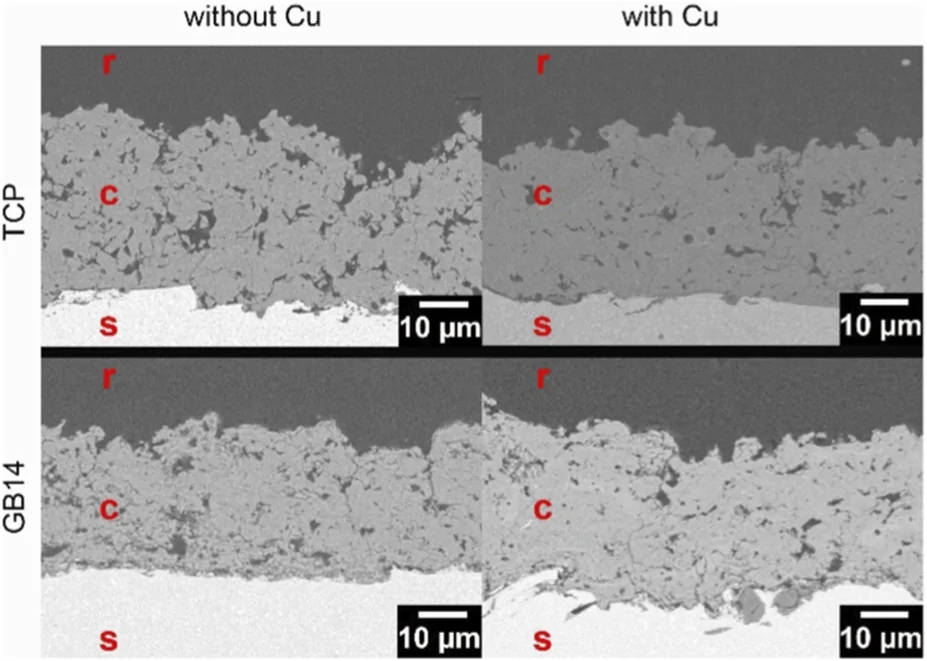
Representative cross-sectional SEM images of the ultra-thin porous CaP coating formulations: r … resin; c … coating; s … substrate. Modified from our previous work (Genchev et al., 2026a) under CC BY 4.0.

**TABLE 3:** Selected physicochemical characteristics of the ultra-thin porous CaP coatings.

Material composition	Ultra-thin CaP Coating		
	Thickness [µm]	Porosity [%]	Surface Roughness Sa [µm]
TCP	31.5 ± 2.4	14.3 ± 1.0	2.4 ± 0.1
TCP/Cu	27.9 ± 2.5	13.1 ± 1.6	2.7 ± 0.1
GB14	28.4 ± 2.5	13.6 ± 1.2	2.6 ± 0.1
GB14/Cu	30.5 ± 2.2	13.4 ± 2.4	3.1 ± 0.4

### 3.2 Peri-implant histological response and cell density

All 19 evaluable specimens showed direct apposition of bone to the implant or residual coating surface without interposed connective tissue, as illustrated by representative sections from all four coating formulations in Figure 6. No histological signs of an inflammatory tissue reaction were observed in the analyzed peri-implant regions. Osteocyte density was descriptively comparable between the pooled β-TCP/all and GB14/all categories (175 ± 79 and 171 ± 50 cells/mm^2^, respectively). Within the β-TCP system, osteocyte density was numerically higher for β-TCP/Cu than for the corresponding Cu-free coating (213 ± 85 vs. 136 ± 57 cells/mm^2^), but the paired difference was not statistically significant (t(4) = −1.98, p_raw_ = 0.119, p_FDR_ = 0.349). Within the four complete GB14 pairs, osteocyte density did not differ significantly between GB14/Cu and GB14 (162 ± 59 vs. 194 ± 38 cells/mm^2^; t(3) = 2.29, p_raw_ = 0.106, p_FDR_ = 0.750). The combined density of surface-associated osteoblasts and osteoclasts also showed no significant Cu-associated difference within either material system (β-TCP: t(4) = −0.84, p_raw_ = 0.450, p_FDR_ = 0.466; GB14: exact Wilcoxon p_raw_ = 0.250, p_FDR_ = 0.750; Table 4; Figure 7).

**FIGURE 6:**
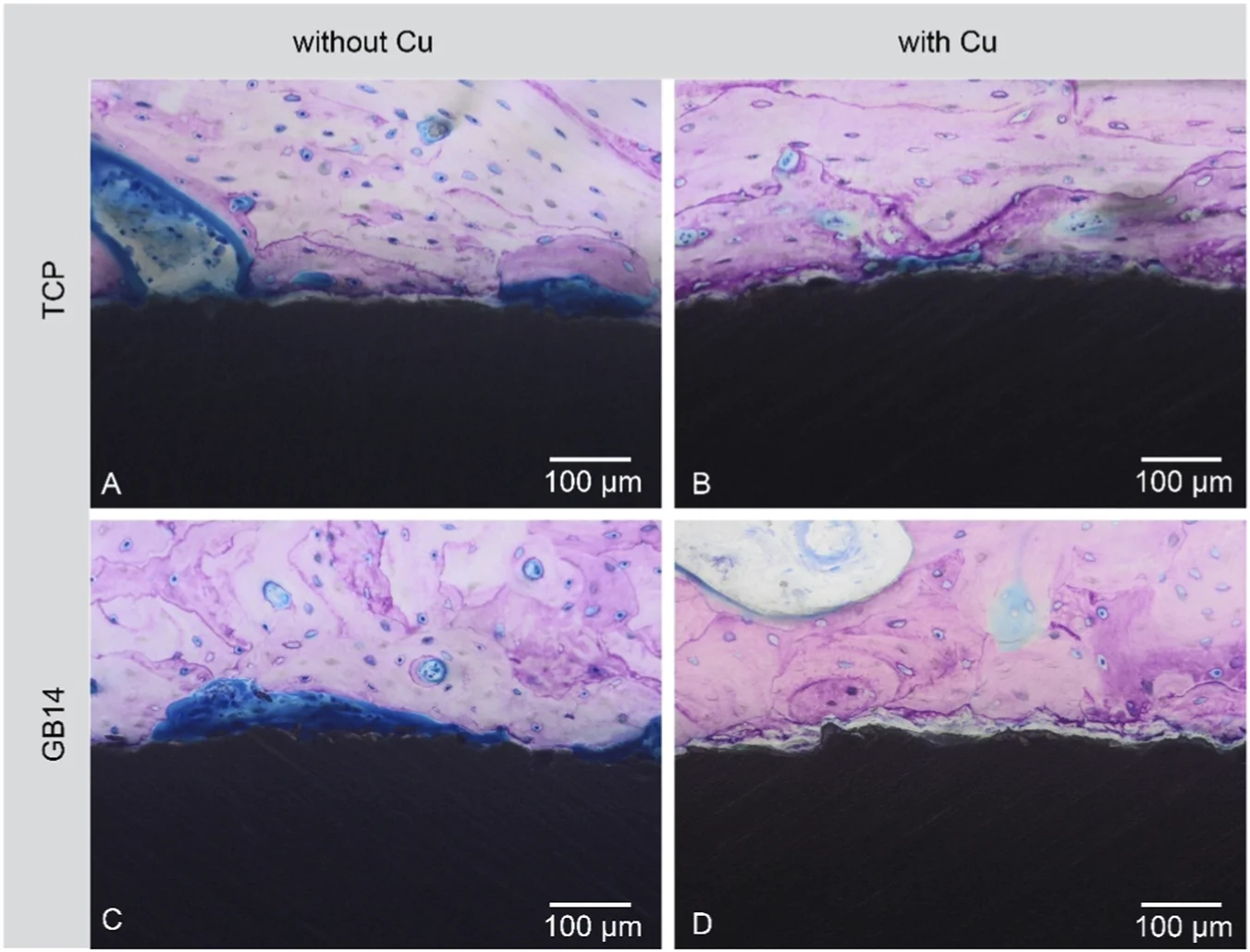
Representative Giemsa-stained histological sections of the bone–implant interface after 24 weeks *in vivo.* **(A)** TCP, **(B)** TCP/Cu, **(C)** GB14, and **(D)** GB14/Cu. The dark region represents the implant, while the adjacent pink-to-purple tissue represents peri-implant bone. Blue-turquoise areas at the implant surface indicate residual CaP coating material. Direct bone apposition to the implant or residual coating surface was observed in all four coating formulations. Scale bars: 100 µm.

**TABLE 4:** Peri-implant osteocyte and surface-associated osteoblast/osteoclast densities.

Group	Osteocyte density [cells/mm^2^]	Osteoblasts/Osteoclasts density [cells/mm^2^]
TCP/all	175 ± 79	50 ± 26
TCP/Cu	213 ± 85	56 ± 33
TCP	136 ± 57	43 ± 20
GB14/all	171 ± 50	57 ± 16
GB14/Cu	162 ± 59	50 ± 9
GB14	179 ± 47	63 ± 18

* For the GB14 group, descriptive values include all five evaluable implants. Paired statistical comparisons were restricted to the four animals with evaluable bilateral specimens; corresponding paired-subset values are reported in the text.

**FIGURE 7:**
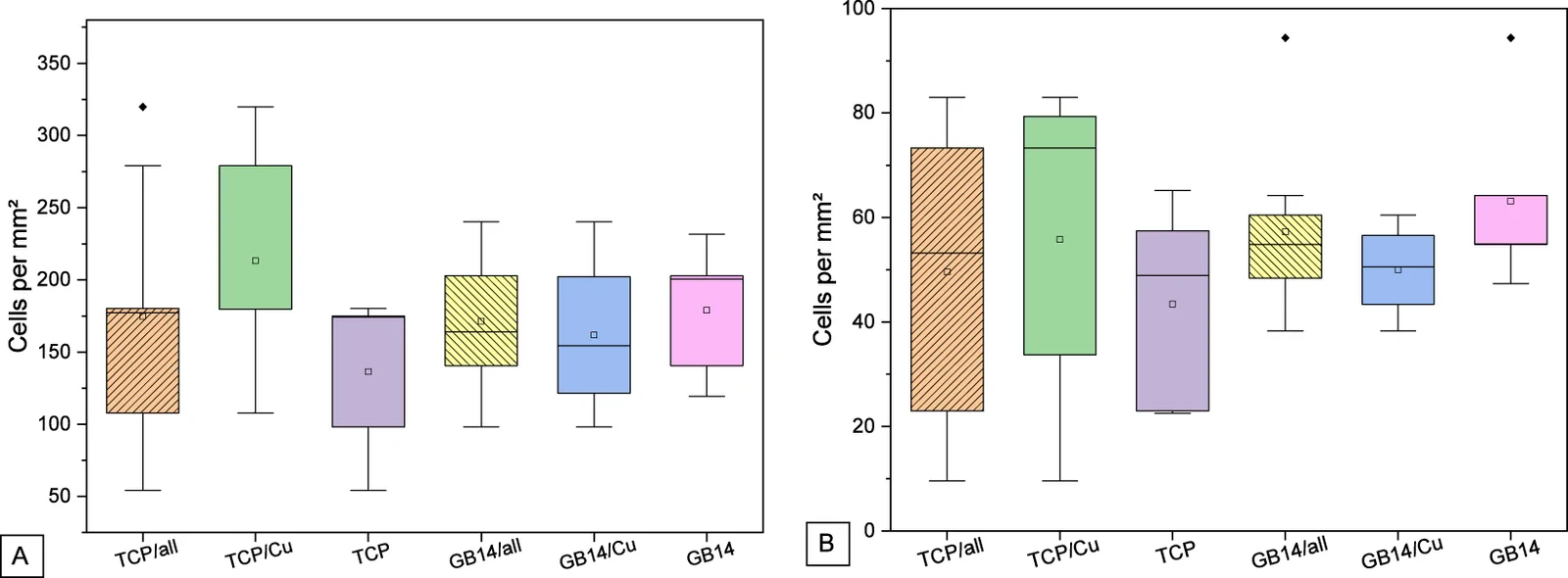
Quantitative histomorphometric analysis of peri-implant bone cell density. **(A)** Osteocyte density. **(B)** Combined surface-associated osteoblast/osteoclast density. Boxes represent the interquartile range, horizontal lines indicate the median, open squares indicate the mean, whiskers extend to the most extreme values within 1.5 times the interquartile range, and individually displayed symbols represent outliers. Statistical comparisons were restricted to copper-functionalized versus corresponding Cu-free coatings within each CaP system using paired-samples tests. No comparison was statistically significant before or after Benjamini–Hochberg false-discovery-rate correction. The pooled β-TCP/all and GB14/all categories are shown for descriptive purposes only and are visually distinguished from the four experimental coating groups.

### 3.3 Bone-implant contact and residual coating coverage

Bone-to-implant contact (BIC) and residual coating coverage were quantified in all 19 evaluable specimens. Mean BIC values ranged from 76.5% ± 14.2% to 86.3% ± 4.6% across the four coating formulations, demonstrating consistently high direct bone contact at the 24-week endpoint (Table 5; Figure 8A). Within the β-TCP system, BIC was numerically higher for β-TCP/Cu than for β-TCP (83.08% ± 4.43% vs. 76.51% ± 14.24%), but the paired difference was not statistically significant (t(4) = −0.95, p_raw_ = 0.394, p_FDR_ = 0.466). Within the four complete GB14 pairs, no significant difference was observed between GB14/Cu and GB14 (86.27% ± 4.57% vs. 79.88% ± 10.14%; t(3) = −0.94, p_raw_ = 0.415, p_FDR_ = 0.887). Residual coating coverage was low across all formulations, ranging from 1.5% ± 1.8% to 6.4% ± 6.8% of the implant perimeter (Table 5; Figure 8B). The residual coating fraction did not differ significantly between β-TCP/Cu and β-TCP (5.26% ± 4.36% vs. 1.51% ± 1.81%; t(4) = −1.98, p_raw_ = 0.119, p_FDR_ = 0.349) or between GB14/Cu and GB14 within the four complete pairs (5.49% ± 2.92% vs. 6.45% ± 7.82%; t(3) = 0.32, p_raw_ = 0.771, p_FDR_ = 0.935). Thus, high BIC and minimal residual coating coverage coexisted at 24 weeks across all investigated ultra-thin porous CaP coating formulations.

**TABLE 5:** Bone-implant contact and residual coating coverage after 24 weeks *in vivo.*

Group	Bone-implant Contact [%]	Residual coating [% of implant perimeter]
TCP/all	79.80 ± 10.53	3.39 ± 3.72
TCP/Cu	83.08 ± 4.43	5.26 ± 4.36
TCP	76.51 ± 14.24	1.51 ± 1.81
GB14/all	83.37 ± 7.57	5.98 ± 5.14
GB14/Cu	86.27 ± 4.57	5.49 ± 2.92
GB14	81.05 ± 9.16	6.38 ± 6.78

* For the GB14 group, descriptive values include all five evaluable implants. Paired statistical comparisons were restricted to the four animals with evaluable bilateral specimens; corresponding paired-subset values are reported in the text.

**FIGURE 8:**
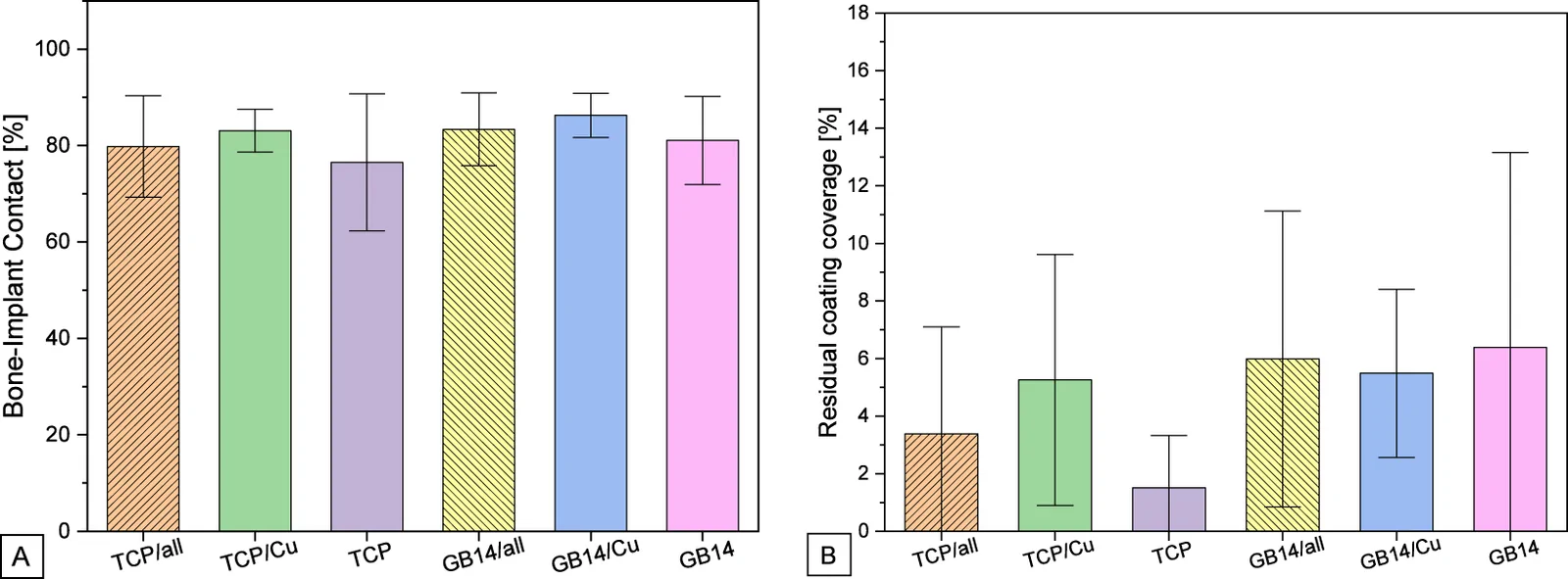
Bone-implant contact and residual coating coverage after 24 weeks *in vivo.* **(A)** Bone-implant contact expressed as a percentage of the total implant perimeter. **(B)** Residual coating coverage expressed as a percentage of the total implant perimeter. Data are presented as mean ± standard deviation. Paired statistical comparisons were performed between copper-functionalized and corresponding copper-free coatings within each CaP system; no significant differences were detected. No comparison was statistically significant before or after Benjamini–Hochberg false-discovery-rate correction. The pooled β-TCP/all and GB14/all categories are shown for descriptive purposes only and are visually distinguished from the four experimental coating groups by hatching.

### 3.4 Peri-implant bone area

Peri-implant bone area was quantified in all 19 evaluable specimens within the predefined 0.5-mm circumferential ROI. Mean bone area ranged from 31.0% ± 19.1% to 56.4% ± 13.2% across the four coating formulations (Table 6; Figure 9). Within the β-TCP system, peri-implant bone area was numerically higher for β-TCP/Cu than for β-TCP (56.40% ± 13.23% vs. 30.97% ± 19.14%); however, the paired difference was not statistically significant (t(4) = −1.92, p_raw_ = 0.127, p_FDR_ = 0.349). Within the four complete GB14 pairs, no significant difference was observed between GB14/Cu and GB14 (39.32% ± 20.03% vs. 51.72% ± 13.05%; t(3) = 0.78, p_raw_ = 0.493, p_FDR_ = 0.887). The descriptively pooled β-TCP/all and GB14/all categories showed similar mean peri-implant bone areas of 43.7% ± 20.5% and 43.8% ± 16.8%, respectively.

**TABLE 6:** Peri-implant bone area within the 0.5-mm circumferential region of interest after 24 weeks *in vivo.*

Group	Peri-implant bone area [%]
TCP/all	43.68 ± 20.50
TCP/Cu	56.40 ± 13.23
TCP	30.97 ± 19.14
GB14/all	43.75 ± 16.76
GB14/Cu	39.32 ± 20.03
GB14	47.30 ± 15.00

* For the GB14 group, descriptive values include all five evaluable implants. The paired statistical comparison was restricted to the four animals with evaluable bilateral specimens; corresponding paired-subset values are reported in the text.

**FIGURE 9:**
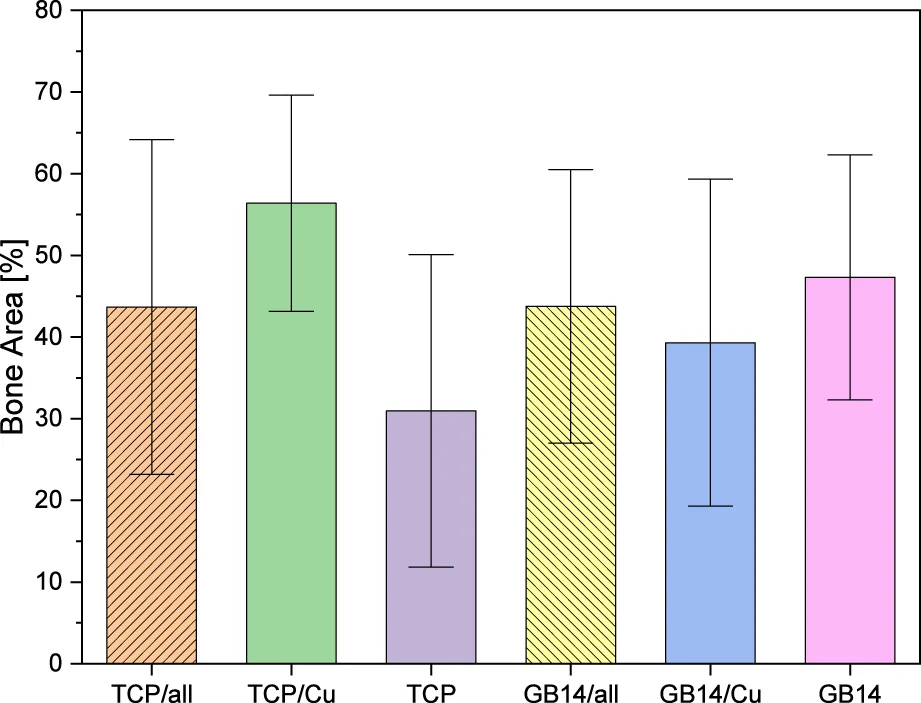
Peri-implant bone area within the 0.5-mm circumferential region of interest after 24 weeks *in vivo.* Data are presented as mean ± standard deviation. Copper-functionalized and corresponding copper-free coatings were compared within each CaP system using paired-samples tests. Neither paired comparison was statistically significant before or after Benjamini–Hochberg false-discovery-rate correction. The pooled β-TCP/all and GB14/all categories are shown for descriptive purposes only and are visually distinguished from the four experimental coating groups by hatching.

### 3.5 Sequential fluorochrome labeling

Fluorochrome-positive area fractions were quantified in 76 ROIs from the 19 evaluable specimens, with four ROI measurements averaged to obtain one specimen-level value for each fluorochrome. Labeling was detected for all four fluorochromes, demonstrating peri-implant bone formation at each investigated labeling time point between 2 and 8 weeks after implantation (Table 7; Figure 10). Within the β-TCP system, no significant Cu-associated difference was detected for tetracycline (p_raw_ = 0.466, p_FDR_ = 0.466), xylenol orange (p_raw_ = 0.290, p_FDR_ = 0.435), calcein (exact Wilcoxon p_raw_ = 0.188, p_FDR_ = 0.349), or alizarin complexone (p_raw_ = 0.194, p_FDR_ = 0.349). Likewise, no significant Cu-associated difference was observed within the GB14 system for tetracycline (p_raw_ = 0.935, p_FDR_ = 0.935), xylenol orange (p_raw_ = 0.896, p_FDR_ = 0.935), calcein (praw = 0.225, p_FDR_ = 0.750), or alizarin complexone (p_raw_ = 0.773, p_FDR_ = 0.935). Because the fluorochromes differed in administered dose and fluorescence characteristics, their absolute area fractions were not compared inferentially and should not be interpreted as quantitative differences in bone formation between labeling time points.

**TABLE 7:** Fluorochrome-positive area fractions (mean ± SD) for tetracycline (T), xylenol orange (X), calcein (C), and alizarin complexone (A), administered at 2, 4, 6, and 8 weeks after implantation, respectively.

Group	Sequential fluorochrome labeling			
	T	X	C	A
TCP/all	0.086 ± 0.026	0.036 ± 0.014	0.068 ± 0.015	0.028 ± 0.006
TCP/Cu	0.093 ± 0.030	0.038 ± 0.017	0.072 ± 0.017	0.030 ± 0.008
TCP	0.078 ± 0.020	0.033 ± 0.011	0.063 ± 0.014	0.025 ± 0.004
GB14/all	0.068 ± 0.036	0.037 ± 0.011	0.058 ± 0.021	0.025 ± 0.012
GB14/Cu	0.068 ± 0.032	0.039 ± 0.012	0.068 ± 0.028	0.023 ± 0.009
GB14	0.068 ± 0.042	0.034 ± 0.011	0.050 ± 0.009	0.027 ± 0.015

* For the GB14 group, descriptive values include all five evaluable Cu-free implants. Paired statistical comparisons were restricted to the four animals with evaluable bilateral specimens.

**FIGURE 10:**
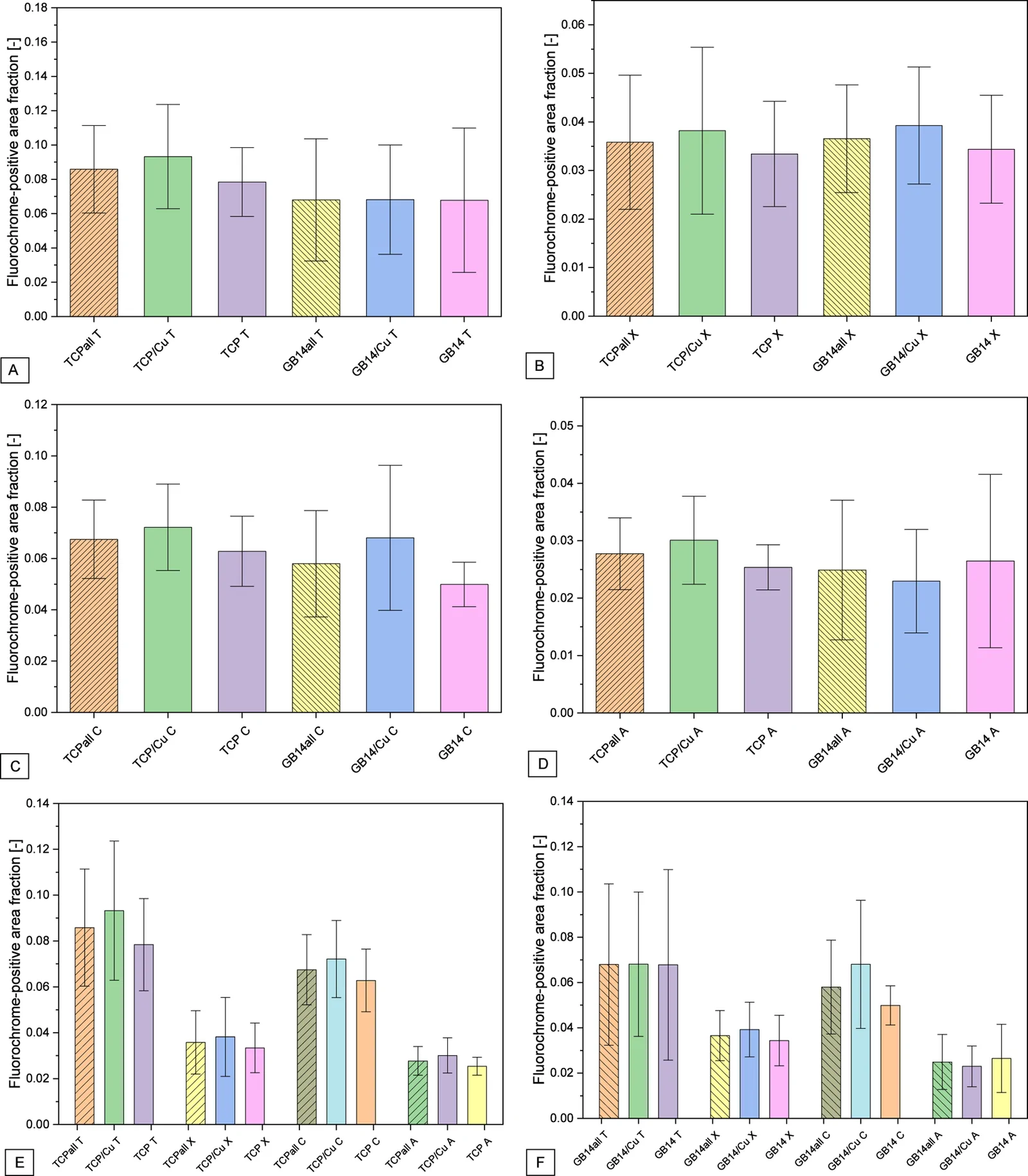
Fluorochrome-positive area fractions in the different coating groups. **(A)** Tetracycline administered at week 2, **(B)** xylenol orange at week 4, **(C)** calcein at week 6, and **(D)** alizarin complexone at week 8. Panels **(E)** and **(F)** provide descriptive summaries of the labeling patterns within the β-TCP and GB14 systems, respectively. Data are presented as mean ± standard deviation. Statistical comparisons were restricted to copper-functionalized versus corresponding Cu-free coatings within the same CaP system and fluorochrome channel. No comparison was statistically significant before or after Benjamini–Hochberg false-discovery-rate correction. Direct quantitative comparisons among the four fluorochromes are not valid because of differences in administered dose and fluorescence characteristics. The pooled β-TCP/all and GB14/all categories are shown for descriptive purposes only and are visually distinguished from the four experimental coating groups by hatching.

## 4 Discussion

### 4.1 Extensive remodeling and high osseointegration coexist after 24 weeks *in vivo*

The principal finding of this study was the coexistence of minimal residual coating coverage and high bone-to-implant contact after 24 weeks *in vivo* across two ultra-thin porous CaP coating systems. Although the initial HVSFS coatings were approximately 28–32 µm thick, only 1.5%–6.4% of the implant perimeter remained covered by histologically identifiable coating material at the endpoint. At the same time, mean BIC values ranged from 76.5% to 86.3%, and direct bone apposition without interposed connective tissue was observed for all coating formulations. These findings demonstrate that extensive remodeling of the investigated ultra-thin coatings was compatible with a highly integrated bone-implant interface at the 24-week endpoint. The simultaneous presence of low residual coating coverage and high BIC is consistent with the hypothesis that these coatings may function as transient osteoconductive interfaces, providing a bioactive surface during early healing without requiring permanent persistence at the implant surface. Reducing coating thickness could theoretically limit the amount of residual ceramic material and some thickness-related risks associated with conventional CaP coatings, including residual stresses, delamination, and particulate release (Heimann, 2017; Wang et al., 1993; Morscher et al., 1998; Schönweger et al., 2020). However, because the present study included neither an uncoated implant nor a conventional-thickness coating as a concurrent control and evaluated coating remnants at only one endpoint, it cannot establish that the observed interface was caused specifically by the reduced coating thickness or that the ultra-thin coatings provide biological or mechanical advantages over conventional coatings. The findings should therefore be interpreted as evidence that extensive loss of histologically detectable coating material was not accompanied by low BIC at 24 weeks, rather than as proof of superiority of the ultra-thin coating design.

### 4.2 Biological remodeling of ultra-thin porous calcium phosphate coatings

Ultra-thin porous bioresorbable CaP coatings are intended to provide a temporary osteoconductive surface during the early phase of implant healing rather than to remain permanently at the bone-implant interface. The minimal residual coating coverage observed after 24 weeks is consistent with extensive remodeling of the ultra-thin porous β-TCP- and GB14-based formulations. Importantly, the low amount of histologically detectable coating material coincided with direct bone apposition and high BIC at the endpoint. In the present study, the term “remodeling” is used operationally to describe the low fraction of histologically identifiable residual coating at the 24-week endpoint and does not establish the biological or mechanical mechanism responsible for coating loss. This extends previous investigations of similarly thin CaP coatings, which demonstrated favorable bone ingrowth and biomechanical fixation, by providing quantitative histological evidence of the interface remaining after extensive coating remodeling (Genchev et al., 2026a; Subkov et al., 2025). Sequential fluorochrome labeling provided complementary information on peri-implant bone formation during the earlier healing period. Fluorochrome-positive bone areas were detected following labeling at 2, 4, 6, and 8 weeks, indicating that bone formation occurred at each investigated time point. Because the four fluorochromes differed in administered dose and fluorescence characteristics, differences in their absolute area fractions cannot be interpreted directly as changes in bone-formation rate over time. Nevertheless, the presence of all four labels demonstrates that the peri-implant bone response was not restricted to a single early labeling time point. Sequential fluorochrome labeling is well established for evaluating the spatial and temporal distribution of mineralizing bone and has been applied in preclinical studies of implant osseointegration and bone regeneration (Pautke et al., 2005; Pautke et al., 2010; Martins et al., 2018). Taken together, the endpoint histomorphometry and sequential-labeling findings are consistent with, but do not directly demonstrate, the investigated ultra-thin porous CaP coatings functioning as transient osteoconductive interfaces. Because coating coverage was quantified only after 24 weeks, the temporal relationship between coating loss and new bone formation cannot be reconstructed. Earlier histological endpoints or longitudinal three-dimensional imaging would be required to determine whether coating removal and bone replacement occurred synchronously.

### 4.3 Copper functionalization within the two ultra-thin porous CaP coating systems

Cu functionalization was not associated with a statistically significant difference in any of the investigated cellular, histomorphometric, or fluorochrome-labeling outcomes within either CaP system, either before or after false-discovery-rate correction. Within the β-TCP system, osteocyte density and peri-implant bone area were numerically higher for the Cu-functionalized coating, but the paired differences did not reach statistical significance. These observations therefore do not provide evidence of a Cu-induced osteogenic or angiogenic effect in the present experiment. The previously reported biomechanical investigation of complementary portions of specimens from the same animal experiment found improved push-out fixation for β-TCP/Cu compared with β-TCP (Pautke et al., 2005; Pautke et al., 2010; Martins et al., 2018). However, the present histological analysis did not statistically corroborate a corresponding Cu-associated difference in osteocyte density, peri-implant bone area, BIC, or residual coating coverage. The numerical increase in peri-implant bone area observed for β-TCP/Cu was also not accompanied by a proportional increase in BIC. These parameters quantify spatially distinct aspects of osseointegration: bone area represents the amount of bone within the 0.5-mm peri-implant ROI, whereas BIC measures the fraction of the implant perimeter in direct contact with bone. Consequently, a greater amount of peri-implant bone does not necessarily result in proportionally greater interfacial contact, particularly when BIC is already high. Copper ions have been associated with osteogenic, angiogenic, and antibacterial effects (Barralet et al., 2009; Pierre et al., 2023; Jacobs et al., 2021). For the same 0.5 wt% supraparticle-functionalized coating formulation used here, previous *in vitro* testing demonstrated cumulative Cu release over 120 h in TRIS buffer of 5.14 mg/L for β-TCP/Cu and 4.61 mg/L for GB14/Cu, with approximately 45%–55% of the measured 120-h release occurring within the first 24 h and a progressively decreasing release rate thereafter (Barralet et al., 2009; Pierre et al., 2023; Jacobs et al., 2021). These data support early Cu availability but do not establish local concentrations or sustained release under *in vivo* conditions. Local Cu release was not measured in the present animal experiment; therefore, any potential early Cu-mediated biological response cannot be reconstructed at the 24-week endpoint, and the findings should not be attributed to sustained Cu stimulation. Moreover, because no stand-alone Cu-only control was included, the study cannot determine whether any potential Cu response depends on the surrounding CaP coating matrix. Importantly, Cu functionalization was not associated with reduced BIC, increased residual coating coverage, or histological signs of an adverse local tissue response in either coating system.

### 4.4 Clinical relevance and future perspectives of ultra-thin porous coatings

The present findings identify ultra-thin porous bioresorbable CaP coatings as a potential surface-design strategy for metallic orthopedic implants. Their initial thickness of approximately 28–32 µm reduces the amount of ceramic material compared with conventional plasma-sprayed CaP layers, while the high BIC observed after 24 weeks demonstrates that extensive remodeling of the investigated coatings was compatible with a highly integrated bone-implant interface. An ultra-thin porous coating may therefore provide a temporary osteoconductive surface during healing without requiring long-term persistence of a comparatively thick ceramic layer. Reducing coating thickness could theoretically limit some thickness-related risks of conventional CaP coatings, including residual stresses, interfacial failure, delamination, and particulate release (Heimann, 2017; Wang et al., 1993; Morscher et al., 1998; Schönweger et al., 2020). However, because no conventional-thickness or uncoated concurrent controls were included, the present study cannot demonstrate a mechanical, biological, or clinical advantage attributable specifically to reduced coating thickness. Such benefits remain a hypothesis requiring direct comparative confirmation.

The coating strategy also addresses only the implant surface and does not resolve substrate-related mechanisms of long-term implant failure. TiAl6Nb7 has an elastic modulus substantially higher than that of cortical bone, and the resulting stiffness mismatch can alter physiological load transfer, contribute to stress shielding, and induce adaptive peri-implant bone resorption (Heimann, 2017; Wang et al., 1993; Morscher et al., 1998; Schönweger et al., 2020). An ultra-thin bioactive coating may support the early bone-implant interface but does not modify the bulk stiffness of the metallic substrate or eliminate the potential for stress shielding. This complementary mechanism was not assessed in the present rabbit model. The HVSFS platform permits incorporation of bioactive components without fundamentally changing the ultra-thin porous coating architecture. In the present study, Cu functionalization was not associated with adverse histological findings, but neither was it associated with statistically significant improvements in the investigated histomorphometric or fluorochrome-labeling outcomes. Future investigations should directly characterize local ion-release kinetics, potential antibacterial efficacy *in vivo,* and the temporal relationship between coating remodeling and peri-implant bone formation. Studies using uncoated, conventional-thickness, and Cu-only controls, additional early endpoints, three-dimensional imaging, clinically relevant implant geometries, larger animal models, and physiological loading conditions will be required to determine the translational potential of this coating concept.

### 4.5 Limitations

Several limitations should be considered when interpreting the present findings. First, the final sample size was limited to four or five evaluable implants per group. Moreover, the original sample-size calculation was driven by the biomechanical endpoint of the overarching animal experiment, whereas the paired Cu-versus-control comparisons reported here were secondary and were not supported by a separate endpoint-specific power calculation. Although the bilateral implantation design enabled paired comparisons of Cu-functionalized and Cu-free coatings within each CaP system, the small number of complete pairs limited statistical power and increased the risk of type II error. Accordingly, the absence of statistically significant differences should not be interpreted as evidence of equivalence between Cu-functionalized and Cu-free coatings. The nine comparisons performed within each material system also increased the potential for type I error; this was addressed using Benjamini–Hochberg false-discovery-rate correction, although multiplicity adjustment further reduces sensitivity in an already small dataset. Three GB14-cohort specimens were excluded because insufficient staining and/or methyl methacrylate embedding artifacts prevented reliable evaluation. Although these exclusions were based on technical image quality rather than the observed biological response, attrition-related bias cannot be excluded.

Second, Cu-functionalized coatings were consistently implanted in the left femoral condyle and Cu-free coatings in the right femoral condyle rather than being randomized between sides. Implant side therefore cannot be separated from Cu functionalization. In addition, the β-TCP and GB14 cohorts underwent surgery in separate sessions. Surgical session and CaP composition are consequently confounded, and comparisons between β-TCP and GB14 were restricted to descriptive presentation. The histological and histomorphometric evaluators were not formally blinded to group allocation, and no formal intra- or inter-observer agreement analysis was performed. Although standardized ROIs, predefined identification criteria, and consistent measurement procedures were applied, observer-related bias cannot be excluded.

Third, no uncoated titanium, conventional-thickness CaP, or stand-alone Cu-only control groups were included. The study therefore cannot determine whether the observed interface differed from uncoated titanium, whether ultra-thin coatings provide advantages over conventional coating thicknesses, or whether any potential Cu response depends on the surrounding CaP matrix. These control groups were omitted to limit additional animal use because uncoated and conventional CaP-coated implants had been investigated in previous related rabbit studies and the present experiment focused on paired comparisons within the two ultra-thin coating systems. Nevertheless, the absence of concurrent controls remains an important limitation of causal interpretation.

Fourth, coating coverage was evaluated at a single endpoint after 24 weeks. The study therefore demonstrates minimal residual histologically identifiable coating at this endpoint but cannot reconstruct the temporal course or precise mechanism of coating removal. The absence of histologically detectable coating material cannot distinguish biological dissolution or cell-mediated resorption from mechanical coating loss during implantation, specimen sectioning, grinding, or subsequent histological processing. Previous *in vitro* testing of the same formulation demonstrated Cu release over 120 h, with an early release phase followed by a decreasing release rate, but local Cu concentrations and release kinetics were not measured *in vivo.* Consequently, neither the duration of Cu exposure nor a relationship between early Cu release and the 24-week histological findings can be established. Furthermore, the selected 0.5 wt% supraparticle loading was based on prior physicochemical and *in vitro* investigations rather than an *in vivo* dose-ranging study.

Fifth, the coatings were sterilized by gamma irradiation at 10 kGy, but phase composition and Cu oxidation state were not assessed specifically before and after sterilization. Potential irradiation-associated alterations of the CaP phases or Cu valence state therefore cannot be excluded and would require dedicated paired X-ray diffraction and X-ray photoelectron spectroscopy analyses. Finally, the assessment was based primarily on two-dimensional histology and histomorphometry. Complementary three-dimensional imaging would provide additional information on the spatial distribution of residual coating and peri-implant bone. Only female rabbits were included, potential sex-specific responses were not assessed, and the model did not address stress shielding or clinically representative long-term loading. Translation to human orthopedic implants will require confirmation using clinically relevant implant geometries, larger animal models, and physiological loading conditions.

## 5 Conclusion

Ultra-thin porous β-TCP- and GB14-based CaP coatings exhibited minimal residual coating coverage together with high osseointegration after 24 weeks in a rabbit femoral condyle model. Across all four coating formulations, only 1.5%–6.4% of the implant perimeter remained covered by histologically identifiable coating material, whereas BIC ranged from 76.5% to 86.3%. Direct bone apposition without interposed connective tissue and the absence of histological signs of an adverse local tissue reaction further supported a favorable bone-implant interface. Fluorochrome labeling demonstrated peri-implant bone formation at each investigated labeling time point between 2 and 8 weeks. Cu functionalization was not associated with statistically significant differences in any of the investigated cellular, histomorphometric, or fluorochrome-labeling outcomes within either CaP system, either before or after false-discovery-rate correction. The coexistence of minimal residual coating coverage and high BIC is consistent with the hypothesis that ultra-thin porous bioresorbable CaP coatings may function as transient osteoconductive interfaces. However, comparative studies incorporating concurrent uncoated, conventional-thickness, and Cu-only controls are required to determine whether this coating architecture provides specific mechanical or biological advantages.

## In memoriam

We dedicate this work to the memory of Anke Bernstein, who initiated this research and tragically passed away in the summer of 2021. The authors would like to thank Sunil Shetty, a native speaker, for spell-checking and grammar correction.

## Data Availability

The raw data supporting the conclusions of this article will be made available by the authors, without undue reservation.
